# Multi-Stream Convolutional Neural Network-Based Wearable, Flexible Bionic Gesture Surface Muscle Feature Extraction and Recognition

**DOI:** 10.3389/fbioe.2022.833793

**Published:** 2022-03-03

**Authors:** Wansu Liu, Biao Lu

**Affiliations:** Information Engineering Department, Suzhou University, Suzhou, China

**Keywords:** multistream convolutional neural networks, wearable flexibility, bionic gestures, surface muscles, feature extraction recognition

## Abstract

Surface electromyographic (sEMG) signals are weak physiological electrical signals, which are highly susceptible to coupling external noise and cause major difficulties in signal acquisition and processing. The study of using sEMG signals to analyze human motion intention mainly involves data preprocessing, feature extraction, and model classification. Feature extraction is an extremely critical part; however, this often involves many manually designed features with specialized domain knowledge, so the experimenter will spend time and effort on feature extraction. To address this problem, deep learning methods that can automatically extract features are applied to the sEMG-based gesture recognition problem, drawing on the success of deep learning for image classification. In this paper, sEMG is captured using a wearable, flexible bionic device, which is simple to operate and highly secure. A multi-stream convolutional neural network algorithm is proposed to enhance the ability of sEMG to characterize hand actions in gesture recognition. The algorithm virtually augments the signal channels by reconstructing the sample structure of the sEMG to provide richer input information for gesture recognition. The methods for noise processing, active segment detection, and feature extraction are investigated, and a basic method for gesture recognition based on the combination of multichannel sEMG signals and inertial signals is proposed. Suitable filters are designed for the common noise in the signal. An improved moving average method based on the valve domain is used to reduce the segmentation error rate caused by the short resting signal time in continuous gesture signals. In this paper, three machine learning algorithms, K-nearest neighbor, linear discriminant method, and multi-stream convolutional neural network, are used for hand action classification experiments, and the effectiveness of the multi-stream convolutional neural network algorithm is demonstrated by comparison of the results. To improve the accuracy of hand action recognition, a final 10 gesture classification accuracy of up to 93.69% was obtained. The separability analysis showed significant differences in the signals of the two cognitive-behavioral tasks when the optimal electrode combination was used. A cross-subject analysis of the test set subjects illustrated that the average correct classification rate using the pervasive electrode combination could reach 93.18%.

## Introduction

In human structure, hands are the most sensitive limb parts, which can sensitively perceive external things and feedback the information touched to the higher nerve center to make timely and precise action responses. Therefore, issues such as how to improve the quality of life of the physically disabled group and enhance the convenience of patients' lives have received attention from all occupations (Chen et al., 2021a). At the same time, the research on prostheses has become an important research direction in the field of rehabilitation medicine. Unlike the early decorative prosthesis, the research on the prosthesis in recent years has tended to be more informative, intelligent, practical, and friendly. With the gradual progress of human–computer interaction research, the research results of intelligent prosthetic hand are emerging, but it has not been able to meet the needs of patients with arm disabilities. The amplitude of the generated surface electromyographic (sEMG) signal will also be large; on the contrary, the amplitude of the generated sEMG signal will be small when the muscle is relaxed. The portability and control flexibility of the smart prosthetic hand need to be improved, so the development of a smart prosthetic hand system has important research significance and value. EMG is the temporal and spatial superposition of motor unit action potentials in numerous muscle fibers and is the source of electrical signals that generate muscle force. EMG is generated by action potentials generated in the motor cortex of the brain that is transmitted through the spinal cord and peripheral nervous system to reach the muscle fibers, which are then low-pass filtered by the skin and finally form an electrical potential field at the surface (Cao and Liu, 2019). The sEMG signal is a noninvasive method of detecting muscle activity as a combined effect of superficial muscle EMG and electrical activity on the nerve trunk at the skin surface, recorded by placing a pair of electrodes near the muscle tissue to be recorded and amplifying the potential difference between the two electrodes through an acquisition system.

Most of the commercially available prostheses are designed according to mechatronics. Although this design can meet the basic needs of patients and assist them in simple grasping movements, there are still many problems to be solved, such as the patient cannot naturally control the prosthesis and can only perform a very small part of the functions of the human hand. There is no obvious distinguishing point between the whole movement changes, and the jitter is also very serious in the continuous phase of the movement, and at the beginning of the movement, there is an irregular wave in the steady phase. In modern society, patients' requirements for prostheses are not only limited to simple functions but also the convenience and practicality of its use (Hassan et al., 2020; Chen et al., 2021b). In recent years, an increasing number of researchers have devoted their attention to this field because of the high social value of intelligent bionic prostheses that can help patients with physical disabilities to participate in life and work with a posture close to that of a normal person.

There are two main ways to interact with the computer by recognizing the user's gesture movements: the first one is achieved by relying on computer vision methods, and the second one is based on sEMG signals for gesture classification. Compared with the method using computer vision for gesture recognition, the method using sEMG signal can avoid the influence of factors such as venue, occlusion, and lighting on the recognition effect, and the user's range of use can be not limited by the camera's line of sight. On the other hand, because the sEMG signal is 30–50 ms ahead of the limb movement, it can reflect the movement tendency and intention more rapidly. Therefore, gesture recognition based on sEMG signals is one of the important ways to realize such perceptual user interfaces.

## Related Work

Since the beginning of the application of EMG in prosthetic control, researchers have invested much effort in solving the classification accuracy problem so that the classification accuracy for sEMG signals can reach more than 90% (Chen et al., 2021c). However, this was done in an ideal laboratory setting, where the types of actions defined are simple, and the number of actions is not large, whereas real-life involves many gestures. However, in real life, many gestures are involved, and if the user is affected by external factors when operating the EMG device, the model may suffer from a sudden drop in recognition accuracy, which is an unstable problem (Tam et al., 2019; Liu et al., 2021a). Therefore, sEMG-based prosthetic control is not yet available for clinical application. There are problems to be solved, both using the latest deep learning methods and using classical pattern recognition methods for gesture classification. The main features of sEMG signals are the following: time-domain features, frequency domain features, time–frequency domain features, and nonlinear features with parametric model analysis (Hu et al., 2019; Su et al., 2020), thereby reducing the influence of the DC offset potential on the sEMG waveform. In addition, during the entire collection process, each experimenter must wear the Myo armband to the same position. In the initial stage, researchers studied the sEMG signal as a function of time, and some statistical features can be obtained from the sEMG signal using simple time-domain analysis methods, and the commonly used time-domain features are wavelength (WL), root mean square (RMS), mean absolute value (MAV), the number of crossing zero points (ZC), etc. Chen et al. collected the sEMG signals of 11 gesture actions from 15 volunteers' data, selected time-domain features such as MAV, ZC, and WL, and performed data whitening preprocessing, which improved the signal classification accuracy by approximately 5% (Jiang et al., 2021a). When the sEMG signals were further investigated, it was found that although the extraction of time-domain features was relatively simple and fast and worked well, there was no relatively obvious physical significance, so the researchers devoted their attention to analyzing the sEMG signals using frequency feature extraction methods (Yang et al., 2019; Holobar and Farina, 2021; Xiao et al., 2021).

Park et al. developed a user-adaptive multilayer convolutional neural network (CNN) model to train the model on sEMG signals using data from six gestures and found that the CNN was much more effective than the support vector machine in both nonadaptive and adaptive modes (Zhao et al., 2022). Yang et al. (2021) designed a high-density electrode with 128 channels and performed experiments on gesture recognition by a classification scheme with deep CNNs. Hao et al. (2021) built a simple CNN with only one convolutional layer, one pooling layer, and two fully connected layers and trained six gestures from seven participants. Liu et al. (2021b) proposed a new model based on deep learning by concatenating a CNN with an RNN, i.e., the output of the CNN is used as the input of the RNN, and the experimental results demonstrated that this concatenation structure improved the accuracy and robustness of recognition. In the sEMG signal classification problem, these deep learning-based methods improve the recognition accuracy compared with the traditional methods and do not involve the processing of feature aspects (Jiang et al., 2021b).

First, due to the similarity of EMG signals of similar gestures, how to detect similarities and not very different movements. Secondly, how to perform gesture recognition on sEMG data with fewer channels, as some prosthetic hands use only two EMG acquisition electrodes due to cost control issues in the commercial field. Then, life involves many gesture movements, so how to maintain high recognition accuracy and reduce the time for model recognition when dealing with many gestures. Next, the mismatch between laboratory data and daily life data, how to keep the recognition accuracy of the model without decreasing is also a problem to be considered. Finally, sEMG signals have attracted the interest of an increased number of researchers due to their extremely high application value in the future, and most of them have made their datasets publicly available, but how to fully exploit the value of these datasets due to the differences in acquisition devices, acquisition gestures, and sampling frequency. In addition to this, there are, of course, many unmentioned pending problems.

## Wearable, Flexible Bionic Gesture Surface Muscle Signal Acquisition

**FIGURE 1:**
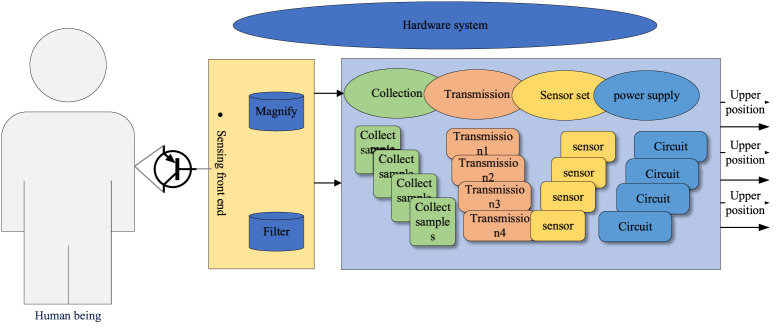
Hardware composition of the acquisition system.

The flexible electrode-based signal acquisition system can acquire sEMG signals from multiple parts of the human body, using a flexible fabric as a substrate on which the electrodes, front-end sensing module, and main control module are all fixed, as shown in Figure 1. Three hundred frames of data were combined into a grayscale image (300 × 16) and then passed into the CNN. The first two layers of the network are convolutional layers. The first convolutional layer contains 64 5 × 5 convolution kernels with a stride of 1, and the second convolutional layer has 64 3 × 3 convolution kernels.

The hardware system includes flexible fabric electrodes, signal sensing front-end, an acquisition processing module, etc. The flexible fabric electrodes are arranged side-by-side on the flexible fabric cuff for multi-channel signal acquisition, and the sEMG signal enters the sensing front-end through the flexible fabric electrodes, and after filtering, amplifying, and other functional circuits, the acquisition processing module performs A/D acquisition and data processing (Wilbanks and Leamy, 2019; Jiang et al., 2019). The acquisition and processing module includes data sampling, wireless transmission, a sensing circuit, and power management. The processed data are transmitted to the PC upper computer by wireless transmission.

The flexible fabric dry electrode material is soft and therefore conforms closely to the skin, and it has the advantage of being soft and comfortable so that it can be used for long-term experiments without causing skin irritation, inflammation, etc. The disadvantage is that the flexible fabric electrodes cannot be fixed by adhesive and require external pressure applied by a fixation device or by an elastic fabric band. This pressure will be transmitted to the skin and cause a deformation of the skin, changing the initial topology of the skin–electrode interface and its electrical properties. Therefore, securing external pressure stability is crucial to suppress motion artifacts. It is more suitable for a real-time control system. It represents the ratio of the total power to the total time in a period, that is, the frequency of the average value of the power spectrum. This feature reflects the intensity of muscle activity and can evaluate the muscle strength of a given hand movement.

Hair on the skin epidermis affects the smoothness of the electrode–skin interface, leading to an unstable contact area, and is highly susceptible to deformation of the capacitive structure of the intermediate interface due to the absence of a buffer zone between the electrode and the skin (Jinqiao et al., 2010). To address these problems, some researchers have introduced conductive foam into the electrode material and structure and compared the physical model effects of foam electrodes and metal electrodes under sliding conditions on skin with hair. In the case of sliding between the skin and the electrode due to forces, the use of conductive foam can effectively adapt to the deformation, reduce the electrode–skin interface gap, and maintain the stability of the interface capacitive topology. Therefore, the use of foam in the preparation of fabric electrodes can enhance the buffering effect.

**FIGURE 2:**
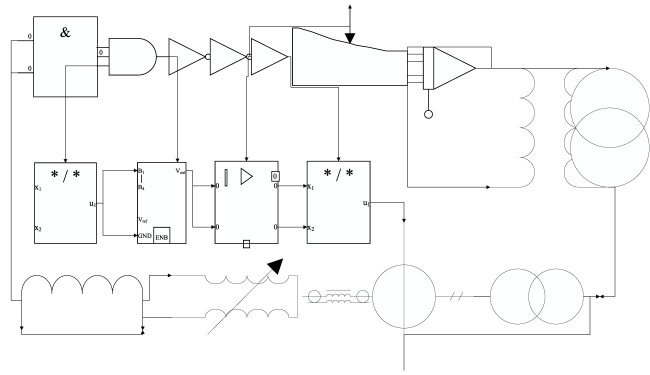
Input-level ESD circuit diagram.

The effective signal amplitude collected from the skin surface by metal or wet electrodes is approximately 0.1∼2 mV, whereas the signal amplitude collected by flexible electrodes will produce approximately 20% attenuation, resulting in the A/D converter of the main control not being able to effectively capture the detailed characteristics of the signal transformation. In addition, it should be noted that the human body generates human static electricity when it moves and accumulates charges, which may discharge when it accumulates to a certain level and cause damage to the post-stage circuit, so the electrostatic protection circuit should be designed at the input stage, and because the post-stage INA321 op-amp is sensitive to electrostatic discharge (ESD), the ESD protection circuit needs to be designed carefully, as shown in Figure 2.

To cope with possible transients and overvoltage situations, it is necessary to use an external protection circuit. Here, a double series switching diode BAV199 is chosen to form a bridge circuit, suitable for low leakage current applications, with a fast-switching time and effective surge voltage suppression. When a high voltage static enters the signal input stage, the bridge directs this high voltage signal to 3.3 V to avoid burning of the rear stage devices. These three models can gradually tend to zero and remain stable within 24 cycles, and they can all fit the training data set well. However, the loss function values of the three models are different, and the loss function of the CNN-long short-term memory (LSTM) model is relatively slow to converge. It also acts as an input level clamp, where the maximum level of the input signal is clamped between 1.5 and 3.3 V. The voltage error is ±0.4% typical at 25°C. It has an internal reference voltage of 2.5 V and a wide range shunt from the cathode to the anode to obtain a fixed reference voltage when output feedback is introduced at the reference.

$$V_{0}=\left(1-R1/R2\right)*2.5V$$ (1)

The sEMG signal has been determined to have a basic amplitude in the range of 100–5,000 μV. It is an extremely weak bioelectric signal also because a certain signal attenuation occurs through the biological tissue and skin after the muscle electrical signal is generated. Analysis of the sEMG signal, which is an AC voltage signal, shows a positive proportional relationship between the magnitude of its amplitude change and the tension of the muscle tissue (Liu et al., 2021c). That is, a large muscle contraction will produce a large amplitude of the sEMG signal, and conversely, a relaxed state of the muscle will produce a small amplitude of the sEMG signal.

In the experiment, the sEMG signal can be decomposed, i.e., the sEMG signal is regarded as consisting of many white noise and sinusoidal waves with zero mean, and the acquired sEMG signal data are arithmetically averaged so that the sum of positive and negative phase values converges to zero.

**FIGURE 3:**
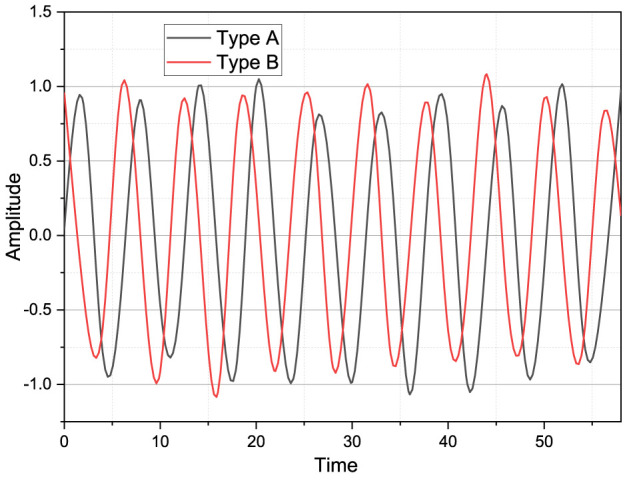
Time-domain signal diagram.

Although there is a simple band-pass filter in the acquisition circuit, the analysis of the original signal, there is still much noise that does not meet our needs, as shown in Figure 3. According to the analysis discussed earlier for the noise introduced in the sEMG signal acquisition experiment, the various noises introduced during the signal acquisition, amplification, and conversion can be removed by a low-pass filter and a 50-Hz trap.

The smooth state of the signal is between 2.5 and 2.7 V, the bottom noise is very severe and not smooth enough, due to the large bottom noise, there is not a very clear one action differentiation point between the whole action changes, and the jitter is very severe during the action duration phase, and there is an irregular fluctuation in the smooth phase at the beginning of the action. In image 3, the whole signal is consistent with the analysis of the low-frequency nature of the sEMG signal, and the signal energy is concentrated between 10 and 200 Hz, but there is a very large component at 50 Hz, which is subject to external industrial frequency interference; after 200 Hz, there are still much irrelevant frequency band components; to obtain a high signal-to-noise ratio of the sEMG signal, the digital filter can be added to remove the irrelevant band signal. To obtain a high S/N signal, a digital filter can be added to remove the irrelevant band signal.

## Analysis of Gesture Surface Muscle Feature Extraction

After acquiring sEMG signals, if the classification is directly taken by machine learning-related algorithms, it does not achieve the expected results, and the reasonable extraction of features from the original data is the top priority. Feature extraction can help extract the most effective information from the original data and achieve the effect of dimensionality reduction, thus helping the classifier to distinguish better the sEMG signals of different actions (Niu et al., 2021). At present, the sEMG signals involve the following four main types of features: time-domain features. The mean absolute value (MAV) is the average magnitude of the absolute value of the sEMG signal within the sliding window, which can reflect the degree of forearm muscle contraction, and is expressed by the following equation. EMG is the superposition of motor unit action potentials in many muscle fibers in time and space and is the source of electrical signals that generate muscle force.

$$MAV=\sqrt{\frac{2}{L}\sqrt{x_{i}}}$$ (2)

In the equation, L is the length of the sliding window, and $x_{i}$ is the value of the *i*th sample point. Other similar features, such as RMS, can also reflect the degree of muscle contraction.

The wavelength length records the sum of the cumulative lengths of the waveforms of the EMG signal and can be expressed simply as the waveform of the signal, expressed by the following equation.

$$WL=\sum_{i=1}^{L}\left|x_{i}+x_{i+1}\right|$$ (3)

This feature shows the complexity of the signal; another feature, average amplitude variation, is more like this feature. The sum of the number of times this feature signal crosses the zero point within the sliding window, which can reflect the variation of different frequency components, is expressed by the following equation.

$$ZC=\sum_{i=1}^{L}\mathrm{sgn}\left|x_{i}x_{i+1}\right|$$ (4)

where to avoid the effect of background noise, a threshold ε can be set so that x>ε for sgn(x)=1 and x≤ε for sgn(x)=0 . The variance feature can represent the energy of the sEMG signal, expressed by the following equation.

$$VAR=\frac{1}{L}\sum_{i=1}^{L}x_{i}^{2}$$ (5)

The idea of the AR model is to use the enhanced average of the previous data with white noise error to predict the subsequent EMG data, expressed by the following equation.

$$x_{k}=\sum_{i=1}^{p}a_{i}x_{k}^{2}-e_{k}$$ (6)

where *p* represents the model order; in this paper, we use AR4; *a*_*i*_ is the coefficient of the model about the EMG signal, *e*_*k*_ is white noise, and *x*_*k*_ is the kth sample point of the sEMG sequence. The main measure is the complexity of the stochasticity of the dynamic system of time series, and the equation is expressed as follows.

$$SampEn\left(x_{i},m,r\right)=\ln\left(\frac{A^{m}\left(r\right)}{B^{m}\left(r\right)}\right)$$ (7)

**FIGURE 4:**
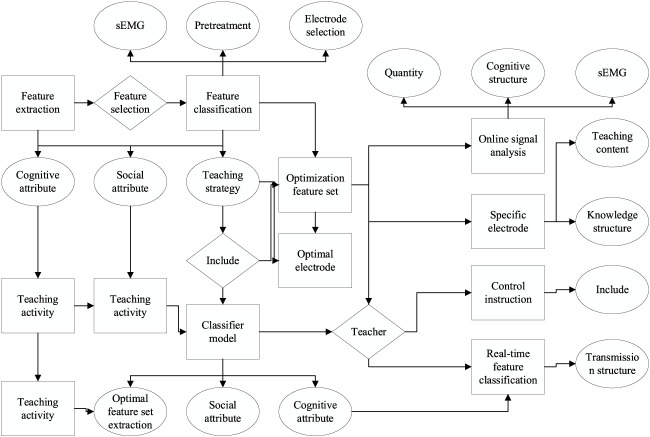
Flow chart of signal feature processing.

To ensure the quality of the sEMG, special attention needs to be paid to the preparation before the sEMG acquisition to reduce the interference of the external environment with the signal. For a while before experimenting, each experimenter was asked not to have too strenuous hand movements to avoid problems such as muscle damage or muscle fatigue before data acquisition. Because the human skin surface has a dense stratum corneum, prolonged exposure to air can make the skin relatively dry, and these conditions can increase the contact impedance between the sEMG electrodes and the skin (Niu et al., 2021). Therefore, the signal acquisition area should be pretreated by first removing the hair from the measured area; then, the experimenter should soak the right small arm in warm water at 36°C for 3 min and gently massage the measured skin area with a scrub to exfoliate the old skin; finally, the area should be disinfected with 75% alcohol. The cleaning and disinfection of the skin can effectively remove the oil and dead skin cells from the skin surface and reduce the impedance between the skin and the electrode, thus reducing the influence of the DC offset potential on the sEMG waveform (Chen et al., 2021a). In addition, each experimenter must wear the Myo armband to the same position throughout the acquisition process and try to make it fit completely to the skin surface, as shown in Figure 4.

In the offline signal analysis stage, the sEMG signal is denoised using infinite impulse response filter, ICA, and MSPCA algorithms; feature extraction includes feature computation in the time domain, frequency domain, and time–frequency domain; single electrode analysis is used to derive the classification correctness ranking and select the two electrodes with the best performance; the feature selection algorithms (EC and DM algorithms) proposed in this chapter are used to extract the feature vectors with a divisibility measure to find the optimal feature subset with generalizability; four machine learning algorithms, including KNN, ANN, RF, and support vector machine, are used to classify the selected feature subset and save the optimal parameters and models of the algorithms. In the real-time signal analysis phase, the preprocessing methods are consistent with the offline analysis except that the signals are collected from only the two selected optimal electrodes, and the signal segmentation method is changed; then, the real-time signal feature vectors are extracted based on the optimal feature subsets, and finally, the features are predicted using the stored classifier models and parameters, and the online control commands are output.

## Multi-Stream Convolutional Neural Network Algorithm Design

This model mainly consists of a multilayer LSTM with CNN, which can learn the spatiotemporal characteristics of the sEMG signal. When a sliding window intercepts a segment of sEMG data (the Elonxi DB dataset uses 300 ms of data), it can be processed as a grayscale map, and the 16 electrodes of the acquisition device are regularly distributed on the forearm, which contains some spatial information inside. At the same time, the data themselves are a time-series signal that varies with time and uses temporal information that exists within it. Based on the EMG signal as a kind of temporal data, the temporal information of the time series can be learned using the long- and short-time memory machine; meanwhile, by merging multiple frames of sEMG data into grayscale maps, the highly abstract spatial features inside the data can be learned using CNNs. Then, by effectively fusing the two features using fully connected layers, the temporal features implicitly inside the data can be learned (Chen et al., 2021b). The recording method is to place a pair of electrodes near the muscle tissue to be recorded and amplify the potential difference between the two electrodes through the acquisition system.

**FIGURE 5:**
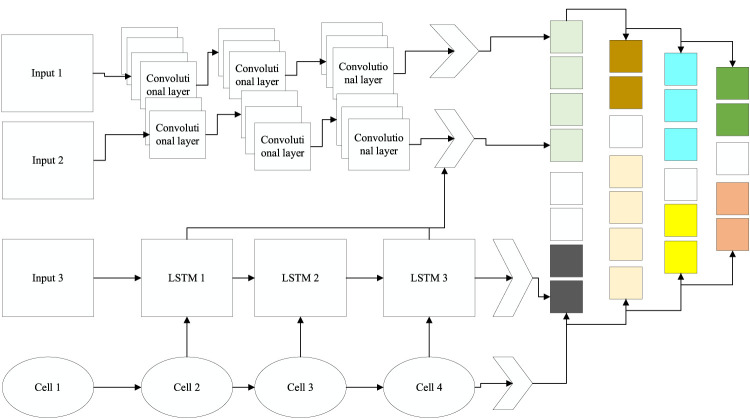
Multi-stream network model.

Starting from the characteristics of the data, this paper proposes a two-stream network model capable of extracting spatiotemporal features, as shown in Figure 5; the model is divided into three main parts: the first part uses CNN to extract the spatial features of the sEMG signal, the second part uses LSTM to extract the temporal features within the signal, and the last part is feature fusion and classification.

Unlike images, EMG signals are very abstract, and no information can be intuitively obtained from them; even so, it is still possible to extract spatial information from the signals that are relevant for classification using a CNN. For the data collected in this paper, each frame represents a 1 × 16 one-dimensional array, and 300 frames of data are combined into a grayscale map (300 × 16), which is then passed into a CNN. The first two layers of the network are convolutional; the first convolutional layer contains 64 5 × 5 convolutional kernels with a step size of 1, and the second convolutional layer has 64 3 × 3 convolutional kernels. Each convolutional layer is followed by a maximum pooling layer that preserves valid information while reducing the data size, followed by two locally connected layers, both with 64 1 × 1 convolutional kernels that enable cross-channel information interaction and increase the nonlinear properties of the model. It has high social value, so increased researchers are also focusing on this field. To extract valid spatial features, a fully connected layer with 256 neurons is provided. The model uses a ReLU nonlinear activation function and is accelerated using batch normalization. To overcome the overfitting problem, the Dropout is set to an activation probability of 0.5. A 256 × 1 spatial feature was obtained for the input sEMG data (300 × 16).

$$f\left(x\right)=\frac{1}{1-e^{-ax}}$$ (8)

where a is the slope parameter of the sigmoid function. Neural networks are commonly used today for pattern recognition classification. Neural networks consist of nodes connected in such a way that each node can represent a particular function called excitation, whereas the connections between the nodes all vary, called weights. With different weights and excitations, the output gets varied accordingly and is suitable for solving a range of nonlinear mapping problems. The algorithm takes as input the sEMG of a hand action recorded in a single experiment and represents the input data as a two-dimensional array S (S ∈ array (m×N)) , where m denotes the number of samples of the input data and N denotes the number of channels of the input data.

The sEMG data are split by rows, i.e., m sEMG samples of size (1 × N) $s_{i}\left(i=1,2,3,...,m\right)$ are obtained, as shown in Eq. 9.

$$S=\left\{s_{1},s_{2},s_{3},...,s_{m}\right\}$$ (9)

At this stage, first, the samples with odd sample labels such as $s_{1},s_{2},s_{3},...,s_{m}$ are reorganized into data block A, as shown in Eq. 10; then, the samples with odd sample labels such as $s_{1},s_{2},s_{3},...,s_{m}$ and other samples with an even number of sample labels are reorganized as data block B, as shown in Eq. 11. Both parts of the reorganized data blocks have dimensions $\left(\frac{m}{2}\times N\right)$ .

$$A=\left\{s_{1},s_{2},s_{3},...,s_{m-1}\right\}$$ (10)

$$B=\left\{s_{2},s_{4},s_{6},...,s_{m}\right\}$$ (11)

The two parts of the reorganized data are directly spliced and fused to obtain a fused data block C of size as $\left(\frac{m}{2}\times N\right)$ , shown in Eq. 12.

$$C=\left\{s_{1},s_{3},s_{5},...,s_{m-1}\right\}$$ (12)

**FIGURE 6:**
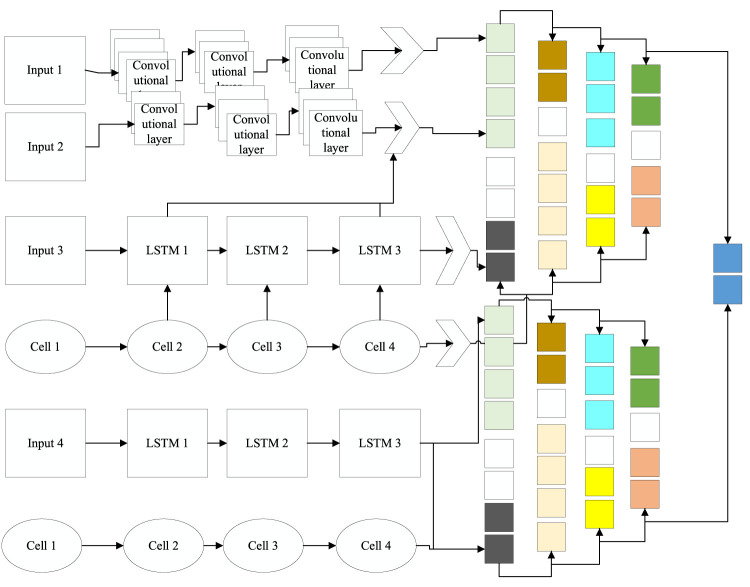
Multifeature fusion model.

The recombination fusion algorithm eventually achieves a multiplication of the number of sEMG channels with a transformation of the size of the data sample from (m × N) to $\left(\frac{m}{2}\times N\right)$ . Although the amount of data before and after the recombination fusion operation is the same, the number of channels of sEMG data is multiplied from N to 2N. In the original input of sEMG samples, taking $s_{1}$ , $s_{2}$ , $s_{3}$ as examples, $s_{1}$ and $s_{2}$ belong to adjacent samples, and $s_{1}$ $s_{3}$ belong to nonadjacent samples. On the one hand, the RCF algorithm combines the nonadjacent samples, and the operation helps to discover the features hidden between the two nonadjacent samples of $s_{1}$ hand $s_{3}$ . On the other hand, after the RCF algorithm, $s_{1}$ and $s_{2}$ are fused into a new sample, which is equivalent to fusing two samples with high similarity for parallel processing, as shown in Figure 6.

In this paper, we use the sliding time window method for data segmentation, which facilitates the calculation and analysis of signal features by obtaining several sEMG sub-data segments. The processing of time windows mainly consists of two methods: adjacent windows and overlapping windows. The adjacent window technique is to split the input sEMG equally according to the settings window length, which causes the processing time of the previous window to be less than the time to obtain the next complete window due to the fast-working speed of the processor, which makes the processor have extra waiting time and thus generates some delay in the control system. Overlapping window technique, i.e., a new window can be fetched for every sliding step of data, reduces the waiting time for window fetching, greatly reduces or avoids the delay in the control system, and is more suitable for application in real-time control systems. It denotes the ratio of total power to total time in a period, i.e., the frequency at which the average value of the power spectrum is located; this feature reflects the intensity of muscle activity, can assess the muscle strength for a given hand movement, and is calculated as follows.

$$MPF=\frac{\int fP\left(f\right)df}{\int P\left(f\right)df}$$ (13)

The median frequency represents the median frequency of the sEMG power spectrum, which is the average of the power of the signal over the periods and is calculated as follows.

$$MF=\frac{1}{2}\sum_{i=1}^{n}P\left(f_{i}\right)$$ (14)

In the convolution process, the convolution kernel is used to perform a regular translational motion in the corresponding region of the input image while completing the extraction of the sample features. Feature extraction means that the convolution kernel performs a convolution operation with the elements in its receptive field and obtains the convolution value of the receptive field, which is usually referred to as the feature map of the input data. To increase the nonlinear segmentation ability of the deep neural network, the activation function is generally introduced after the convolution operation, and among the commonly used activation functions, the sigmoid function and tanh function belong to the saturated activation function, and the relu function belongs to the unsaturated activation function, and the function expressions are shown in Table 1.

**TABLE 1:** Expressions for the activation function.

Activation function	Formula
Sigmoid	$f\left(x\right)=\frac{1}{1+e^{-x}}$
Tanh	$f\left(x\right)=\frac{e^{x}-e^{-x}}{e^{x}+e^{-x}}$
Relu	f(x)=max(0,x)

Before the use of activation functions, each layer of the network operation was only a matrix multiplication operation, and activation functions can add a nonlinear factor to the output data, so it is important to choose the right activation function. The slope of the sigmoid and tanh activation function images is large when close to the zero region, followed by a saturation of the data at both ends of the image when the derivative is approximately equal to zero (Chen et al., 2021a). This type of activation function is prone to gradient disappearance and information loss, which is not conducive to the learning of deep neural networks, so the ReLU activation function is generally chosen in hand-action recognition. Compared with the other two functions, the ReLU activation function sets the output of some neurons to zero, reducing the number of parameters for network learning, which is simple to apply, less computationally intensive, and less prone to gradient disappearance. The influence of factors such as occlusion and illumination on the recognition effect and the user's range of use cannot be restricted by the camera's line of sight.

In a CNN, several convolutional and pooling layers are followed by two or more fully connected layers, which combine the previously acquired features. The locally valid information acquired by the convolutional and pooling layers is tiled, i.e., converted to one-dimensional data, before the fully connected operation is performed. The former is a linear weighted summation of the input data with the weight parameters, which is simply the multiplication of each neuron node with the corresponding weight coefficients W plus the bias b.

## Results of Surface Muscle Signal Acquisition

**FIGURE 7:**
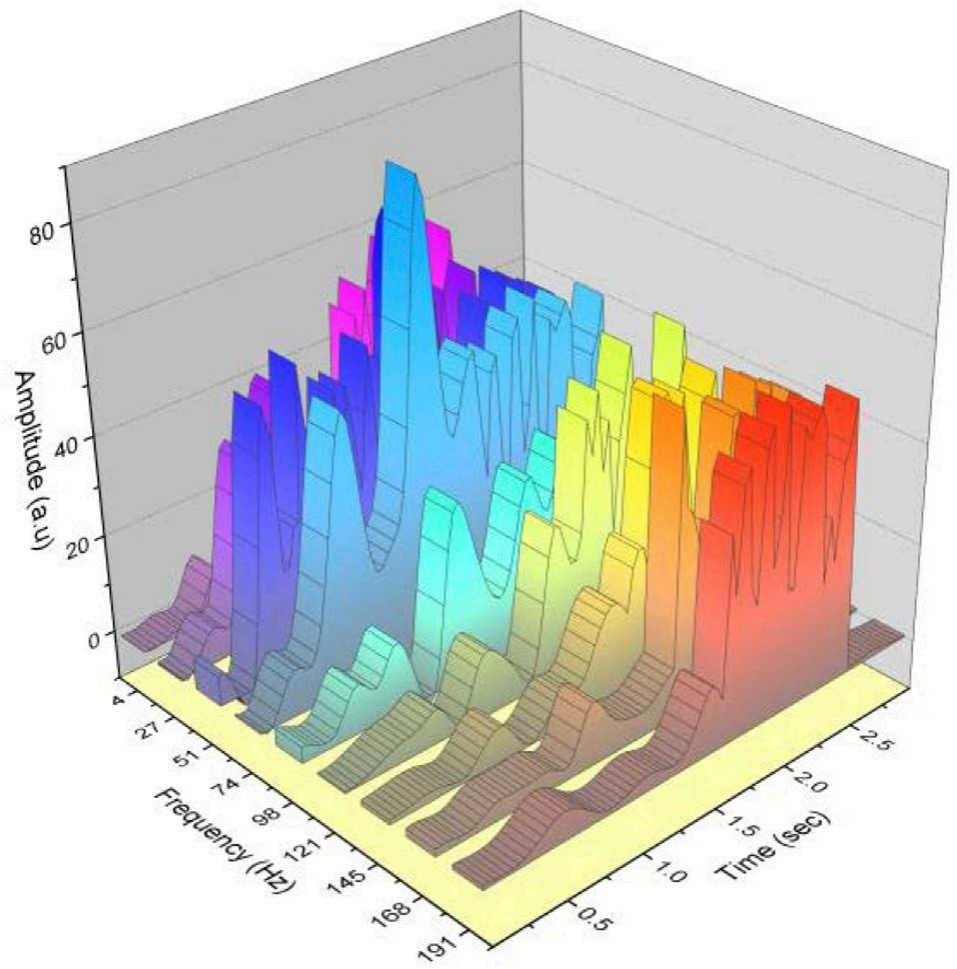
Comparison of muscle signal filtering for deep squatting movements in the time domain.

The acquired signal is passed through a seventh-order Butterworth band-pass filter and a third-order 50-Hz trap, and the obtained signal is compared with the signal without processing and analyzed in both the time-domain–frequency domain and the time domain variation, which are shown in Figure 7.

The filtering in the program will rectify the signal and will cause the balanced resting state in the center of the signal to move to the 0-V position, whereas on the whole image, it can be seen that the original severe bottom noise has been significantly reduced, the whole signal becomes clear, and the changes in amplitude are smaller than the original, and at the beginning of the action, it is clearer than the original unprocessed signal. In the time domain, the processing of the signal has been greatly improved. As can be seen from the comparison chart on the frequency domain in Figure 7, the signal through the filter, the band-pass filtering, and trapping effect in the frequency domain is significant; in the 50-Hz industrial frequency interference position, the interference noise is obviously removed, in the frequency band after 150 Hz also attenuated, and in the frequency after 200 Hz filtered. A/D acquisition and data processing are performed by the acquisition and processing module. Among them, the acquisition and processing module includes functions such as data sampling, wireless transmission, sensing circuit, and power management. The processed data are transmitted to the host computer on the PC side by wireless transmission.

**FIGURE 8:**
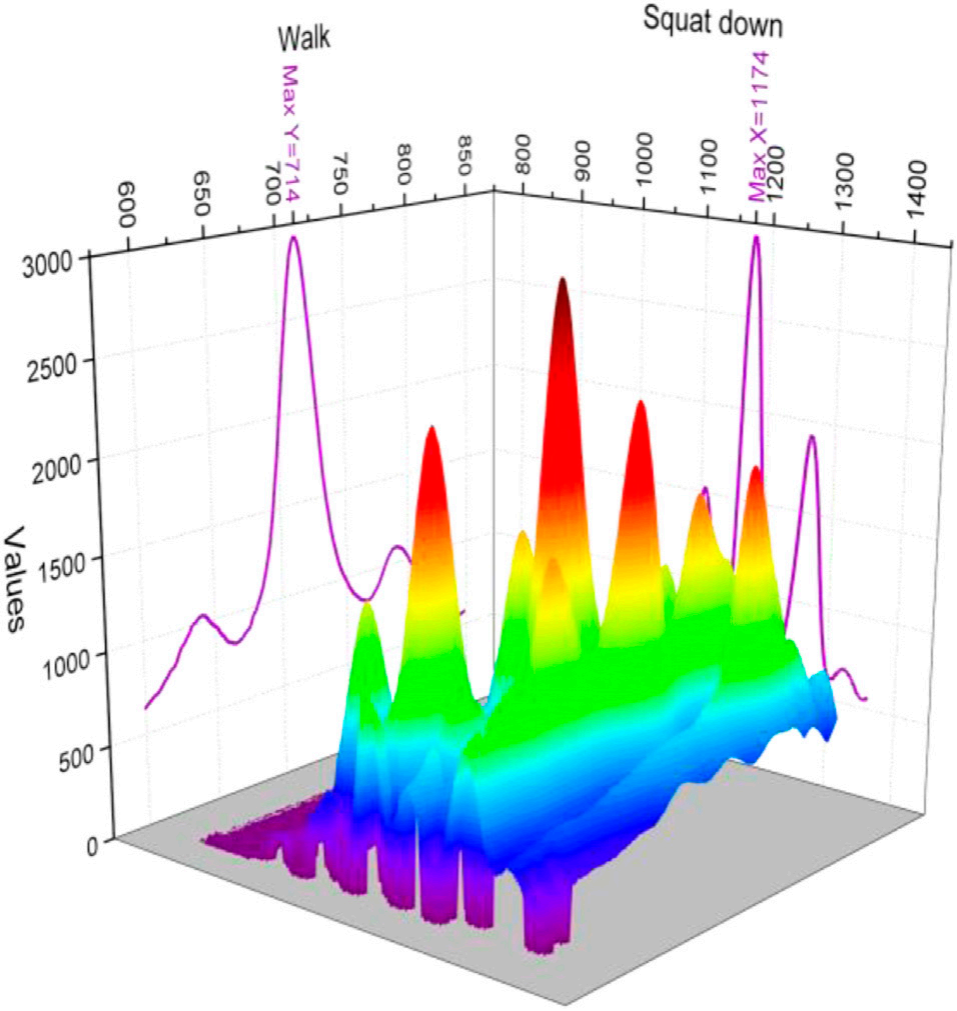
Standard deviation of the broad fascial tensor for the four movements.

In the whole experimental process, the relevant information is recorded timely and accurately, and the preset experimental steps are strictly followed to carry out as far as possible to avoid other interference on the experimental data, the impact. The extracted signal values, after filtering and preprocessing, with a preset program for the extraction of time-domain features, drawing the eigenvalue image, are more intuitive. Considering is for the determination of the pattern of typical actions, for each action in the selection of the most representative of action for display and processing. The four actions are shown specifically in Figure 8.

From Figure 8, we can see that when performing the movements between the four movements, sitting and squatting as similar squatting type movements, the movements were consistent in the peak of the standard deviation, whereas the peak of getting up was smaller, and the peak of walking movements was the smallest, if only the peak was used for judgment, it could be distinguished between the walking and getting up movements, whereas squatting and sitting movements that were similar could not be distinguished effectively.

In the standard deviation of walking and squatting, the trend of change is close, but in the value of deep squatting, it is much larger than the new princess, and with sitting, the value is similar; the trend of change is also somewhat similar, numerically for walking up and down can be identified, whereas sitting and squatting can not be effectively distinguished. The RMS value can be seen in the numerical determination only; in the four movements, only sit down and get up are similar, the squat is the largest, the walk is the smallest, whereas sitting down and getting up have different trends in the RMS value.

Ensuring the stability of external pressure is the key to suppressing motion artifacts. Both standard deviation and RMS values have significant differences in peak values on the rectus femoris, and the trend of change during the movement is more pronounced in the RMS value. Having greater research value, in comparison, the RMS value is less calculated than the standard deviation, so comparing the two, it is concluded that: The RMS value has more significant characteristics in the differentiation of the action, and this difference, with strong signal specificity, can be used as a representative on the time-domain features as an important element of the feature vector for subsequent pattern recognition.

## Feature Extraction Recognition Results

**FIGURE 9:**
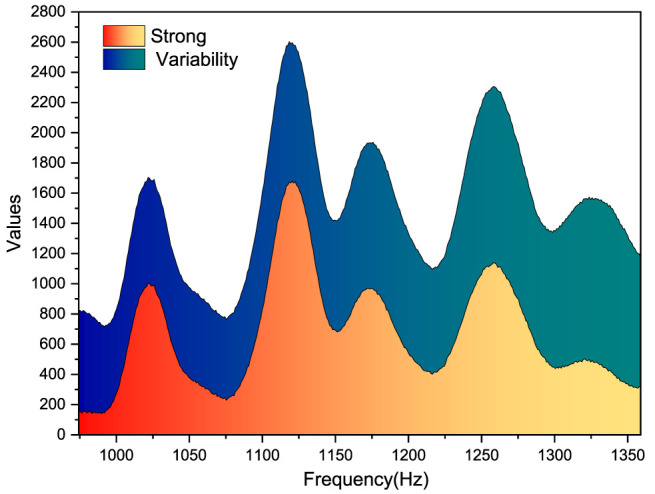
Power spectrum of the broad fascial tensor muscle for the four movements.

From Figure 9, it can be seen that the overall trend of the rectus femoris power spectra of the four movements in progress is similar, all increasing first, with some jitter in the work duration process, with the power peaks being the largest for the deep squat and the smallest for the walk, whereas the rise and sit are similar, so it can be initially seen that a distinction can be made in the power spectra between the deep squat and the walk, whereas there is no strong variability between the rise and sit. Even the rectus femoris area, where the power generation is obvious, could not be differentiated on the power spectrum between the two movements of the rise and sit, and the power spectrum of the four movements of the broad fascia tensor muscle was compared next.

For Figure 9, the power spectra of the broad fascia tensor for the four movements remain largely consistent with the power spectra of the rectus femoris, and the power spectra of the walk have decreased in value but can still be distinguished from the get-up and sit down, with the maximum still being the deep squat. The stability of the topological structure of the interface capacitance is maintained. Therefore, the use of foam in the preparation of fabric electrodes can enhance the cushioning effect. The power spectra of the rise and sit were consistent with similar patterns of change and similar peak values on the images. Therefore, it can be initially seen that the power spectrum can differentiate between deep squat and walking, whereas there is no strong difference between rising and setting, and further calculation should be done to extract the median frequency and average power frequency in the power spectrum to analyze the difference between the movements to see if the further judgment can be made on the movements.

**FIGURE 10:**
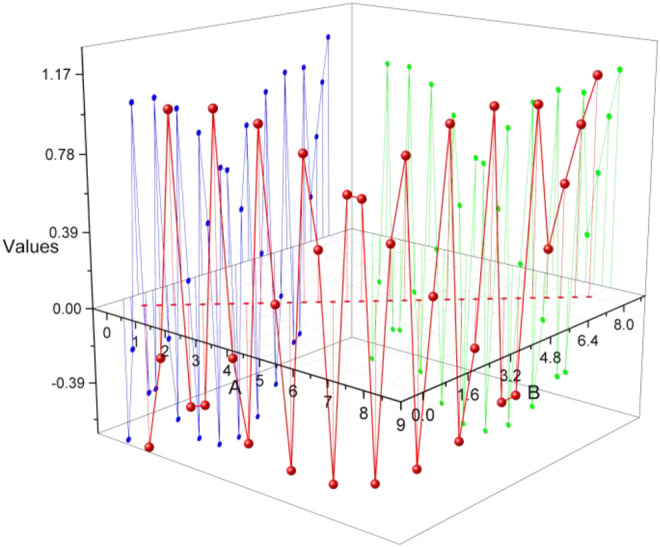
Results of SEMG signal feature extraction in the lower computer.

From the collected data calculated median frequency and average power frequency, the analysis can be concluded that after the more obvious differences can be seen in the power spectrum, it further becomes the median frequency and average power frequency; comparing the values can be seen that walking in the median frequency average frequency is obviously greater than the remaining three movements, whereas sitting down second, squatting third, and rising up last, but this is because the upper and lower limits of each movement change. However, because the upper and lower limits of each action vary greatly, there will be more crossover data, and it is not good to distinguish the four types of actions purely by median frequency, whereas in the average power, although the data of get up and squat are similar, the values of deep squat are slightly larger than get up, and this difference characteristic can assist the time-domain features for action identification, but again, due to the large fluctuation of upper and lower limits, it is not effective to distinguish the four types of actions, as shown in Figure 10.

Although the SEMG signal acquired by the front-end circuit is amplified and filtered after conditioning, the design of the desired filter performance of the board-level analog filter is difficult to achieve due to factors such as board design, signal transmission, external interference, and analog filter structure, so a digital filter needs to be designed for secondary processing. The effective signal amplitude collected from the skin surface through the metal or wet electrode is between 0.1 and 2 mV, and the signal amplitude collected by the flexible electrode will produce approximately 20% attenuation, leading to the main controlled A/D converter that cannot effectively collect the detailed characteristics of the signal conversion. The time domain and frequency domain characteristics of the original SEMG signal are shown in Figure 10. The signal processing at the online upper computer side involves three main parts: de-baselining drift, digital high-pass filter, and digital trap filter. The de-baseline drift is to remove the slow low-frequency drift noise due to temperature drift and human breathing. The digital high-pass filter is to compensate for the poor loading capacity of the hardware analog high-pass filter and the lack of steep filter edges; the digital trap filter is to eliminate industrial frequency noise interference signals.

## Analysis of Algorithm Performance Results

**FIGURE 11:**
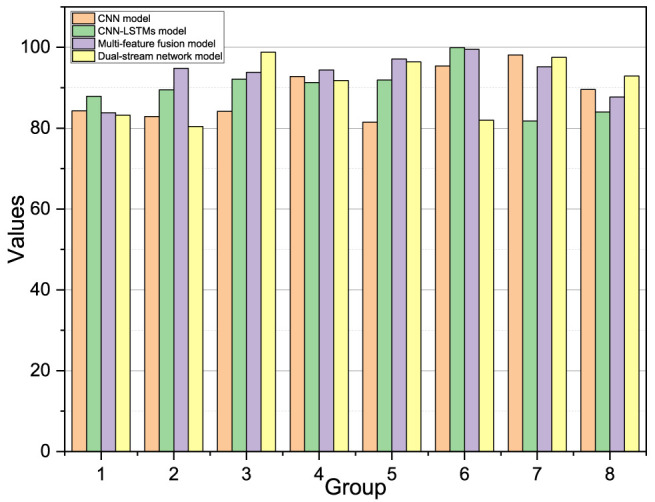
Average accuracy of eight sets of experiments for four models.

From Figure 11, the CNN-LSTM model type, the multifeature fusion model, and the dual-stream network model used in this paper possess higher recognition results on the intersubject experiment compared with the model using CNN alone. This is because the three models designed in this paper have more parameters, higher model complexity, can fit the EMG data better, and take the means of overfitting, so these three models will have better recognition accuracy. Compared with the multifeature fusion model and the two-stream network model, the CNN-LSTM model first uses the CNN module to extract the features within the signal, at which point most of its internal temporal features have been lost or disrupted, and then, the LSTM module can only extract less temporal information, so the model does not have stronger expressive power. Compared with the two-stream network model, the multifeature fusion model incorporates information from the traditional features but instead distracts from the model, and as the parameters increase, the higher complexity of the model does not necessarily have a positive impact.

From the experimental results, the three models designed in this paper have better results in all eight experimenters, although the recognition accuracy has slightly lowered compared with the other groups because the sixth participant is female and the other participants in the training set are male. This experiment demonstrates that the three models designed in this paper perform in addition to better recognition accuracy, with the dual-stream network model that can fuse spatiotemporal features having the highest gesture classification accuracy.

**FIGURE 12:**
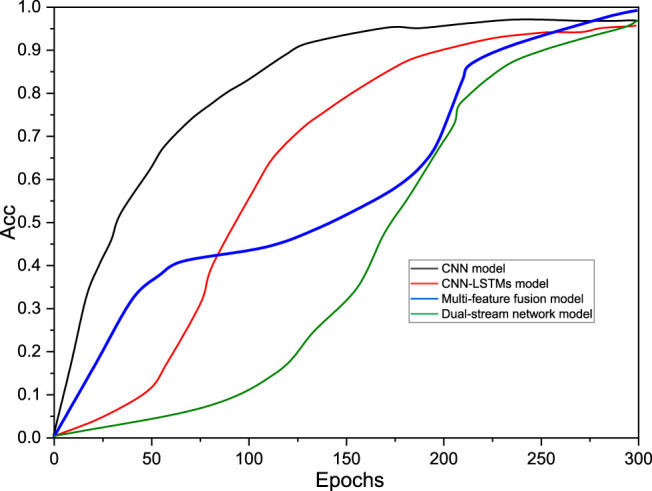
Variation of accuracy for the four models.

To further analyze the degree of strengths and weaknesses of each network model, the data derived from the experiments conducted in the first set for the three networks are illustrated. Figure 12 shows the convergence of the loss function values of the three models over different iterations and the change in the recognition rate of the three models for the training set over 24 iterations. From these two, all three models gradually converge to zero and remain smooth within 24 iterations, and all fit the training dataset better. The loss function of the CNN-LSTM model is relatively slow to converge and does not produce strong fluctuations in the fit to the training dataset; the convergence speed of the multifeature fusion model is in the middle; the two-stream network model can converge faster and is more stable, and the fit to the training data in the late training period can converge faster and is more stable, and the fit to the training data is stable in the later stage of training.

In summary, all four models proposed in this paper can achieve good recognition accuracy on the test set and can also achieve a good fit to the training set, and the loss function can also remain relatively stable while approaching zero. Among them, after each comparison experiment, the two-stream network model is the best model among these three models, and its loss function can approach the optimization limit as soon as possible while ensuring the optimal recognition accuracy, and it can maintain good stability.

## Conclusion

The existing research on pattern recognition-based methods for sEMG signals has focused on new feature design, different feature combinations with a selection of existing classifiers. For designing new features, the researcher has already done much design, and then, discovering new effective features becomes tough and can consume much time and effort. Feature combinations also require more effort from the experimenters to explore, and feature sets that are more appropriate in one scene are likely to become inappropriate when applied in another scene. In deep learning-based gesture classification, most studies have utilized the myoelectric signal converted to two-dimensional data and used CNNs to extract highly abstract spatial features within the data. However, sEMG signals are a type of temporal signal that must contain temporal features within them that are beneficial for signal classification. Many studies have focused only on the spatial features of sEMG signals and ignored the temporal features. Here, a double series switching diode BAV199 is selected to form the bridge circuit, which is suitable for low leakage current applications, has a fast-switching time, and can effectively suppress the surge voltage.

Pattern recognition-based approach *versus* deep learning-based approach. The principles of feature extraction and classifiers of traditional methods are also detailed, and the key techniques and principles of deep learning are also illustrated. Then, pattern recognition-based methods involve feature design and feature combination, which is often the part that will consume much of the researcher's effort, and the models using only CNNs cannot fully extract all the valid information of the EMG signal. Therefore, three classification models are proposed in this paper: the CNN-LSTM model, the multifeature fusion model, and the dual-stream network model. The experimental results prove that the models proposed in this paper are all superior to the models using only CNNs on the self-collected datasets, among which the dual-flow network model, which can extract the spatiotemporal features of sEMG signals, is the most superior. All three models proposed in this paper can better improve the recognition accuracy of sEMG signals, among which the dual-flow network model that can extract spatiotemporal features of sEMG signals has the best performance and can better distinguish the differences of similar gestures, which has certain research value, and this structure of using a CNN to extract spatial features and using long and short time memory machine to extract spatial features can also be used as a researcher in the same field of reference direction.

## Data Availability

The raw data supporting the conclusion of this article will be made available by the authors, without undue reservation.
